# Monocyte surface expression of Fcγ receptor RI (CD64), a biomarker reflecting type-I interferon levels in systemic lupus erythematosus

**DOI:** 10.1186/ar3017

**Published:** 2010-05-18

**Authors:** Yi Li, Pui Y Lee, Erinn S Kellner, Matthew Paulus, Juliana Switanek, Yuan Xu, Haoyang Zhuang, Eric S Sobel, Mark S Segal, Minoru Satoh, Westley H Reeves

**Affiliations:** 1 Division of Rheumatology & Clinical Immunology, University of Florida, 1600 SW Archer Rd, Gainesville, FL 32610-0221, USA; 2 Division of Nephrology, Hypertension and Transplantation, University of Florida, 1600 SW Archer Rd, Gainesville, FL 32610-0221, USA

## Abstract

**Introduction:**

More than half of systemic lupus erythematosus (SLE) patients show evidence of excess type I interferon (IFN-I) production, a phenotype associated with renal disease and certain autoantibodies. However, detection of IFN-I proteins in serum is unreliable, and the measurement of interferon-stimulated gene (ISG) expression is expensive and time consuming. The aim of this study was to identify a surrogate marker for IFN-I activity in clinical samples for monitoring disease activity and response to therapy.

**Methods:**

Monocyte surface expression of Fcγ receptors (FcγRs), chemokine receptors, and activation markers were analyzed with flow cytometry in whole blood from patients with SLE and healthy controls. FcγR expression also was measured in peripheral blood mononuclear cells (PBMCs) from healthy controls cultured with Toll-like receptor (TLR) agonists, cytokines, or serum from SLE patients. Expression of ISGs was analyzed with real-time PCR.

**Results:**

Circulating CD14^+ ^monocytes from SLE patients showed increased surface expression of FcγRI (CD64). The mean fluorescent intensity of CD64 staining correlated highly with the ISG expression (MX1, IFI44, and Ly6E). *In vitro*, IFN-I as well as TLR7 and TLR9 agonists, induced CD64 expression on monocytes from healthy controls. Exposure of monocytes from healthy controls to SLE sera also upregulated the expression of CD64 in an IFN-I-dependent manner. Decreased CD64 expression was observed concomitant with the reduction of ISG expression after high-dose corticosteroid therapy.

**Conclusions:**

Expression of CD64 on circulating monocytes is IFN-I inducible and highly correlated with ISG expression. Flow-cytometry analysis of CD64 expression on circulating monocytes is a convenient and rapid approach for estimating IFN-I levels in SLE patients.

## Introduction

It has become increasingly clear that the autoantibody responses characteristic of systemic lupus erythematosus (SLE), such as anti-double-stranded (ds) DNA and anti-Sm, as well as certain clinical manifestations, notably lupus nephritis, are linked to the overproduction of type I interferon (IFN-I) [1-5]. The importance of IFN-I in autoimmunity is evident in the association between autoimmune manifestations and IFN-α treatment in some patients with hepatitis C infection, malignant carcinoid syndrome, or chronic myelogenous leukemia [6-8]. A positive fluorescent antinuclear antibody test can be found in up to 22% of patients treated with IFN-α [6], and the onset of SLE, autoimmune (Hashimoto) thyroiditis, autoimmune hemolytic anemia, rheumatoid arthritis, vasculitis, and other autoimmune diseases has been reported after IFN-α therapy [7,9,10].

More than half of SLE patients display abnormally high expression of a group of IFN-I-stimulated genes (ISGs), a feature associated with active disease, renal involvement, and the production of autoantibodies against DNA-protein and RNA-protein autoantigens [1-5]. Because of the inherent insensitivity and unreliability of measuring IFN-I protein levels in the blood, the level of ISG transcript expression in peripheral blood mononuclear cells (PBMCs) is frequently used as a measure of IFN-I activity [1-5]. However, these assays are costly and time consuming. Flow cytometry may afford a rapid and less expensive means of evaluating IFN-I levels than RNA-based methods. The objective of this study was to identify proteins encoded by ISGs expressed on the cell surface that can be used clinically to evaluate IFN-I levels in SLE. We show that CD64 (Fcγ receptor I) expression on monocytes can be used to assess IFN-I levels rapidly and reliably in clinical samples and may be well suited to monitoring disease activity and response to therapy.

## Materials and methods

### Patients and controls

SLE patients were selected based on fulfilling four or more of the revised 1982 American College of Rheumatology criteria [11]. One hundred eight SLE patients and 83 healthy controls were studied. Demographic data, clinical manifestations, medication use, and laboratory measurements are summarized in Table 1. Four patients received high-dose methylprednisolone (1 g IV daily for 3 days) for active renal disease. This study was approved by the University of Florida Institutional Review Board, and all subjects provided informed consent.

**Table 1:** Demographics, laboratory, and clinical characteristics of subjects

	Controls (n = 83)	SLE (n = 108)
Demographics		
Female (%)	93	94
Mean age (years)	36	38
Race/ethnicity (%)		
African-American	35	36
White	32	40
Others	33	24
Disease duration (years)	-	12.1 ± 0.7
ACR criteria (mean)	-	6.2 ± 0.2
		
Serum markers		
C3 (mg/dL)	123.4 ± 5.7	95.4 ± 5.5
C4 (mg/dL)	25.7 ± 3.5	19.7 ± 1.5
hsCRP (mg/dL)	1.4 [1.1-4.4]	5.7 [4.1-7.1]
		
SLE manifestations^a^(%)		
CNS	-	18
Skin	-	63
Joint	-	84
Serositis	-	34
Anti-dsDNA	-	61
Anti-Sm	-	45
Anti-phospholipid	-	50
		
Medication use (%)		
Prednisone	-	51
Mean dose (mg/day)		15.5
Antimalarials	-	70
Cytotoxic agents^b^	-	21
Statins	-	18
ACE inhibitors	-	48

^a^Presence of specific manifestations at any point during the course of disease.

^b^Cytotoxic agents included cyclophosphamide, mofetil mycophenolate, azathioprine, and methotrexate.

ACR, American College of Rheumatology; C3, C4, complement 3 and complement 4; hs-CRP, high sensitivity C-reactive protein; SLE, systemic lupus erythematosus.

### Isolation of RNA from PBMCs

Blood was collected in PAXgene tubes, and total RNA was isolated by using the PAXgene RNA kit (Qiagen, Valencia, CA, USA). RNA (1 to 2 μg per sample) was treated with DNase I (Invitrogen) to remove genomic DNA and reverse transcribed to cDNA by using Superscript II First-Strand Synthesis System (Invitrogen) for RT-PCR. RNA and cDNA samples were stored at -70°C until used.

### Real-time quantitative PCR

Expression levels of three IFN-I-inducible genes, myxoma resistant gene-1 (MX1), interferon-inducible protein 44 (IFI44), and Ly6E, were determined in duplicate by real-time PCR (SYBR Green Core Reagent Kit, Applied Biosystems, Foster City, CA, USA). As demonstrated in previous studies, these ISGs are robust markers of IFN-I upregulation associated with SLE [3-5]. Gene expression was normalized to β-actin, and expression relative to the sample with the lowest expression was calculated by using the 2^-ΔΔCt ^method [12]. Amplification conditions were as follows: 95°C for 10 minutes, followed by 45 cycles of denaturation at 94°C for 15 seconds, annealing at 60°C for 25 seconds, and elongation at 72°C for 25 seconds. After final extension at 72°C for 10 minutes, a melting-curve analysis was performed to ensure specificity of the products. For each ISG, a score was calculated based on the number of standard deviations above or below the mean expression of the designated control group [13]. The ISG index was determined based on the average of individual ISG scores (that is, (MX1 + Ly6E + IFI44)/3) [3,13]. Primers were as follows: β-actin forward 5'-TCC CTG GAG AAG AGC TAC GA-3'; reverse 5'-AGC ACT GTG TTG GCG TAC A-3'; MX1 forward 5'-CAC GAA GAG GCA GCG GGA TCG-3', reverse 5'-CCT TGC CTC TCC ACT TAT CTT C-3'; Ly6E forward 5'-AGG CTG CTT TGG TTT GTG AC-3', reverse 5'-AGC AGG AGA AGC ACA TCA GC-3'; and IFI44 forward 5'-CTG GGG CTG AGT GAG AAA GA-3', reverse 5'-AGC GAT GGG GAA TCA ATG TA-3'; CXCL9 forward 5'-TGC TGG TTC TGA TTG GAG TG3', reverse 5'-TCA ATT TTC TCG CAG GAA GG-3'; CD14 forward 5'-ATT TGG TGG CAG GAG ATC AA-3', reverse 5'-GCT TCC AGG CTT CAC ACT TG-3'; CD16 forward 5'-ACA GGT GCC AGA CAA ACC TC-3', reverse 5'-TTC CAG CTG TGA CAC CTC AG-3'; CD32 forward 5'-TTC AAG GCC AAC AAC AAT GA-3', reverse 5'-GGA GAA GGT GGG ATC CAA AT-3'; CD64 forward 5'-GTG TCA TGC GTG GAA GGA TA-3', reverse 5'-GCA CTG GAG CTG GAA ATA GC-3'; CCR2 forward 5'-ATC TCC GCC TTC ACT TTC TG-3', reverse 5'-AAT GCG TCC TTG TTC AAT CC-3'; CCL2 forward 5'-CTG CTC ATA GCA GCC ACC TT-3', reverse 5'-TCC TGA ACC CAC TTC TGC TT-3'; CX3CR1, forward 5'-GAC TGG CAG ATC CAG AGG TT-3', reverse 5'-ACC AAC AAA TTT CCC ACC AG-3'; CX3CL1, forward 5'-GGC TCC GAT ATC TCT GTC GT-3', reverse 5'-CTG CAC GTG ATG TTG CAT TT-3'.

### Cell-surface staining

Fluorescently tagged antibodies were from BD Bioscience (San Diego, CA, USA), unless otherwise indicated. Heparinized whole blood (100 μl) was stained with phycoerythrin (PE)-conjugated anti-CD64 (clone X54-5/7.1.1), PerCP-conjugated anti-CD14 (clone MΦP9), fluorescein isothiocyanate (FITC)-conjugated anti-CD16 (clone 3G8), allophycocyanin (APC)-conjugated anti-CD32 (clone FLI8.26), PerCP- anti-HLA-II (clone L243), APC-conjugated anti-CD62L (clone DREG 56, eBioscience, San Diego, CA, USA), APC-conjugated anti-CCR2 (clone 48607, R&D Systems, Minneapolis, MN, USA), PE-anti-CX3CR1(clone 2A9-1, MBL International Corporation, Woburn, MA, USA), for 20 minutes in the dark. After erythrocyte lysis, cells were washed with PBS/1%BSA/0.01% NaN_3 _and fixed in 2% paraformaldehyde PBS. For dendritic cell characterization, cells were stained with Lin-FITC (a cocktail of anti-CD3, -CD14, -CD16, -CD19, -CD20, and -CD56), anti-CD123-PE (clone 9F5), anti-HLA-DR-PerCP, and anti-CD11c-APC. For T- and B-cell characterization, anti-CD3-FITC (clone UCHT1, eBioscience) and -CD19-PerCP (clone SJ25C1) were used. Cells (10^5^) were analyzed by using a FACSCalibur flow cytometer and CellQuest software (Becton Dickinson, Mountain View, CA, USA). Gates were set around monocytes based on their forward/sideward light-scatter pattern and CD14 expression; lymphocyte gates were set based on forward/sideward light scatter. CD16, CD32, and CD64 expression levels were expressed as the geometric mean fluorescence intensity (MFI). Data were analyzed by using FCS Express 2.0 (De Novo Software, Ontario, Canada). Preliminary studies indicated that CD64 expression on monocytes is stable for at least 24 hours after blood collection (our unpublished observations). Intracellular protein expression of CCL2 was determined by using anti-human CCL2 (clone 5D3-F7; BD Pharmingen) as described previously [14].

### Culture of PBMCs with cytokines or serum

Human PBMCs were isolated from healthy donor cells by Ficoll density-gradient centrifugation. PBMC were plated on 24-well plates (10^6 ^cells per well) in DMEM supplemented with 10% fetal bovine serum, 20 mmol/L L-glutamine, 100 IU/ml penicillin, and 100 μg/ml streptomycin. Cytokines were from BD Bioscience, unless otherwise indicated. Cells were incubated for 19 hours at 37°C in medium containing 25% serum from either SLE patients (n = 65) or healthy controls (n = 44), or in the presence of recombinant human IFN-α (4 ng/ml; PBL Biomedical, Piscataway, NJ, USA), TLR4 agonist (ultrapure *E. coli *lipopolysaccharide (LPS), 1 μg/ml, Sigma-Aldrich), TLR7 agonist (R848, 1 μg/ml; Invivogen, San Diego, CA, USA), or TLR9 agonist (CpG-A ODN2216, 10 ng/ml; Invivogen). The concentration of TLR ligands used in these experiments was determined based on our preliminary studies using PBMCs from healthy controls. For each TLR ligand, the lowest concentration that induced maximal CD64 expression on control monocytes after 19 hours was selected (data not shown). In some experiments, the soluble viral IFN-I antagonist B18R (from vaccinia virus Western Reserve strain, 0.1 μg/ml; eBioscience, San Diego, CA, USA), anti-human IFN-γ (2 μg/ml), anti-human IL-12 (2 μg/ml), or isotype control mouse IgG1 (Biolegend, San Diego, CA, USA) was added 1 hour before stimulation with TLR agonists. Flow cytometry was performed immediately after incubation. For the analysis of serum-induced CD64 expression, ΔMFI was calculated by subtracting baseline CD64 MFI from the MFI of CD64 expression after incubation with serum from healthy controls (n = 44) or SLE patients (n = 65). A positive ΔMFI indicates an upregulation of CD64 expression compared with the baseline levels. All serum samples were stored at -80°C before these experiments. For real-time quantitative PCR studies, PBMCs (10^6 ^cells/well) were treated with PBS or recombinant IFN-α (4 ng/ml), and RNA isolation was performed after 6 hours. Average fold-differences in mRNA expression in PBMCs treated with PBS or IFN-α (n = 4 per group) were determined with real-time PCR, whereas changes in protein levels on monocytes were measured with flow cytometry. Positive values denote increased expression after IFN-α treatment compared with PBS treatment.

### Statistical analysis

Differences between disease groups and normal controls were evaluated by using Student's two-tailed *t *test unless the data were not normally distributed, in which case the Mann-Whitney *U *test was used. Changes in CD64 and ISG expression levels after high-dose corticosteroid therapy were assessed by using the paired Student *t *test. Correlation coefficients were calculated by using Spearman's rank correlation. Data are presented as mean ± SEM. Analyses were performed by using Prism software, version 4.0 (GraphPad Software, San Diego, CA, USA). A *P *value of < 0.05 was considered significant.

## Results

### CD64 expression on monocytes is upregulated in SLE patients

**Figure 1:**
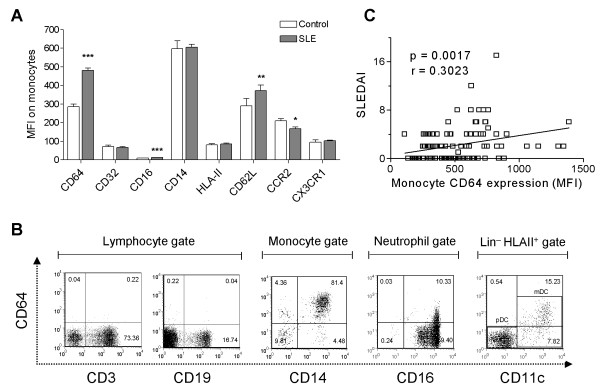
**CD64 expression on monocytes is increased in SLE**. **(a) **Flow-cytometry analysis of monocyte markers in SLE patients (n = 108) and healthy controls (n = 83). Bars represent the average mean fluorescent intensity on CD14^+ ^monocytes, and error bars denote standard error. *P < 0.05; **P < 0.01; ***P < 0.001. **(b) **Representative flow cytometry of CD64 expression on peripheral blood cells from a lupus patient, including CD3^+ ^T cells, CD19^+ ^B cells, CD14^+ ^monocytes, CD16^+ ^neutrophils, and CD11c^+ ^dendritic cells (primarily myeloid dendritic cells). Lymphocytes, monocytes, and neutrophils were gated based on their forward/sideward scatter characteristics. Dendritic cells were first gated on Lin^-^, HLA-DR^+ ^cells, and then further identified as myeloid dendritic cells (CD123^-^, CD11c^+^) with flow cytometry. **(c) **Bivariate analysis of CD64 expression on monocytes (MFI, determined with flow cytometry) and SLEDAI (n = 108). Correlation coefficient was calculated by using Spearman's rank correlation (*P *= 0.0017; *r *= 0.301).

To identify potential biomarkers associated with SLE, we first analyzed a panel of monocyte surface markers, including CD14, Fc receptors (CD16/FcγRIII, CD32/FcγRII, CD64/FcγRI), activation markers (class II MHC, CD62L/L-selectin), and chemokine receptors (CCR2, CX3CR1). Comparing circulating SLE with healthy control monocytes, the greatest difference was found in the surface expression of CD64 (MFI 480.9 ± 12.0 versus 285.6 ± 13.9; *P *< 0.0001; Student's *t *test, Figure 1a). Expression of CD16 and CD62L was elevated less dramatically (MFI 12.8 ± 0.3 versus 10.2 ± 0.6, *P *< 0.0001; 371.7 ± 30.4 versus 291.1 ± 38.4, *P *< 0.001, respectively, Student's *t *test, Figure 1a). Surface expression of CCR2, a marker of the "inflammatory" monocyte subset, was slightly reduced in lupus patients, and no difference was found in the expression of CX3CR1, a chemokine receptor preferentially expressed by "residential" monocytes [15]. In both healthy controls and SLE patients, CD64 was expressed constitutively on circulating CD14^+ ^monocytes and CD11c^+ ^myeloid dendritic cells (MDCs) (Figure 1b) In contrast, CD64 was expressed at low levels on peripheral blood CD16^+ ^neutrophils, and no expression was found on CD3^+ ^T cells or CD19^+ ^B cells (Figure 1b). CD64 expression on monocytes correlated with disease activity, as measured by SLEDAI (Figure 1c). Elevated CD64 expression also was associated photosensitivity, skin manifestations, renal involvement, pericarditis, and hematologic abnormalities. In addition, the presence of anti-dsDNA and anti-Sm autoantibodies, but not anti-phospholipid antibodies, was linked to increased CD64 expression (Table 2). Consistent with our previous observations [14], the use of conventional lupus medications, including oral corticosteroids, antimalarials, and cytotoxic agents, did not affect CD64 expression (Table 2). Demographic data, including age, gender, race, and the number of years since diagnosis, also were not associated with the levels of CD64 expression (data not shown).

**Table 2:** Comparisons of CD64 expression (mean fluorescence intensity) with disease manifestations and medication use

	Yes	No	*P *value
Disease manifestations			
Skin	495.2 ± 15.9	455.5 ± 14.1	0.0423
Photosensitivity	502.3 ± 16.0	446.9 ± 14.4	0.0103
Joint	479.3 ± 13.2	456.4 ± 17.2	0.3481
Renal	499.2 ± 14.4	448.7 ± 15.4	0.0172
CNS	463.3 ± 24.8	475.1 ± 11.6	0.7092
Serositis	488.9 ± 18.9	462.6 ± 12.5	0.2294
Pleurisy	499.0 ± 23.3	463.1 ± 11.9	0.1328
Pericarditis	558.8 ± 26.8	450.7 ± 10.9	< 0.0001
Hematologic abnormalities	495.0 ± 15.3	446.2 ± 14.2	0.0226
Anti-dsDNA	507.0 ± 13.8	439.5 ± 17.9	0.0030
Anti-Sm	489.9 ± 12.0	399.7 ± 23.7	0.0022
Anti-phospholipid	476.0 ± 15.7	476.2 ± 15.4	0.9946
Medications			
Corticosteroids	528.5 ± 20.4	512.9 ± 24.9	0.6409
Antimalarials	510.4 ± 18.1	556.0 ± 31.9	0.1967
Cytotoxic agents	503.9 ± 16.7	557.9 ± 22.6	0.1080
Statins	517.1 ± 37.9	483.9 ± 16.7	0.4460

Differences between groups were analyzed by using Student's *t *test. A *P *value < 0.05 is considered statistically significant. Hematologic abnormalities include autoimmune hemolytic anemia, WBC <4,000/μL, absolute lymphocyte count <1,500/μL, and platelets <100,000/μL.

### CD64 expression is IFN inducible and correlates with the interferon signature

**Figure 2:**
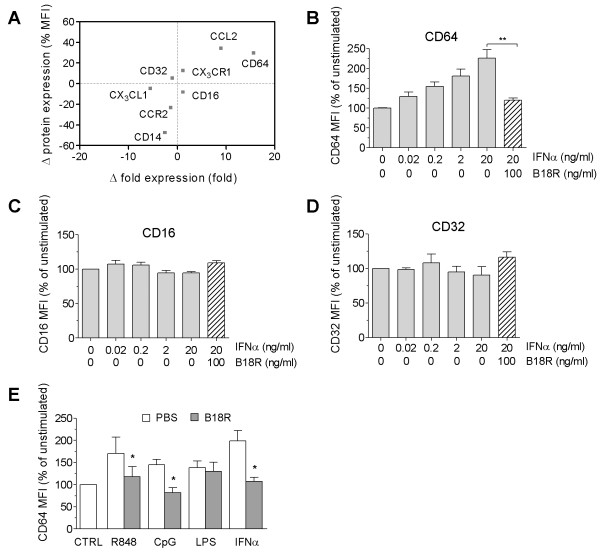
**CD64 expression is inducible by IFN-I and TLR agonists**. **(a) **Effects of recombinant IFN-α (4 ng/ml) on mRNA and protein expression of monocyte markers. Mean differences (fold increase/decrease versus control) in mRNA expression in PBMCs treated with PBS or IFN-α (n = 4 per group) were determined with real-time PCR. Changes in cell-surface protein levels on monocytes (MFI) were measured with flow cytometry. Positive values denote increased expression after IFN-α treatment compared with controls (PBS). **(b** through **d)** Dose-response analysis of CD64 (b), CD16 (c), and CD32 (d) expression *in vitro *on IFN-α-stimulated monocytes from healthy controls. In some groups, B18R (0.1 μg/ml) was added 1 hour before stimulation with IFN-α. **(e) **Induction of monocyte CD64 surface expression by R848 (1 μg/ml), CpG-DNA (10 ng/ml), LPS (1 μg/ml), or IFN-α (4 ng/ml) in the presence/absence of B18R (added 1 hour before stimulation). Flow-cytometry analysis was performed 19 hours after stimulation. Values represent the mean ± SEM from three independent experiments. *P < 0.05 (Student's *t *test).

Because previous microarray studies using RNA from PBMCs identified CD64 as an ISG [2,16], we examined whether exogenous IFN-I can induce CD64 expression on monocytes. Among the monocyte surface markers tested, CD64 was consistently upregulated at the mRNA and protein levels after stimulation with IFN-α (Figure 2a). In line with the observations of others [1,2,17], IFN-α also increased the expression of the chemokine CCL2 (also known as monocyte chemoattractant protein-1; MCP-1), but not its receptor CCR2. CD14 expression, conversely, was reduced after IFN-α treatment (Figure 2a), possibly because of initiation of DC differentiation from monocytes *in vitro *[18].

Detailed analysis of CD64 with flow cytometry showed that the addition of IFN-α to monocytes from healthy donors stimulated its surface expression in a dose-dependent manner. This effect was blocked completely by pretreatment with the soluble vaccinia virus IFN-I antagonist B18R (Figure 2b). In contrast, surface expression of other FcγRs (CD16 and CD32) was unaffected by IFN-α treatment (Figure 2c and 2d).

Recent studies suggest that activation of Toll-like receptor (TLR) 7 and TLR9 may be upstream of the aberrant production of IFN-I in SLE [19-23]. Similar to direct stimulation with IFN-α, treatment with the TLR7 ligand R848 or the TLR9 ligand ODN2216 both induced monocyte surface expression of CD64, an effect that was abolished by pretreatment with B18R (Figure 2e). In contrast, the low level of CD64 upregulation in response to the TLR4 ligand LPS was unaffected by IFN-I blockade. These observations demonstrated that CD64 expression on monocytes is inducible by direct IFN-I stimulation or by TLR7/9 agonists, which elicit IFN-I production.

**Figure 3:**
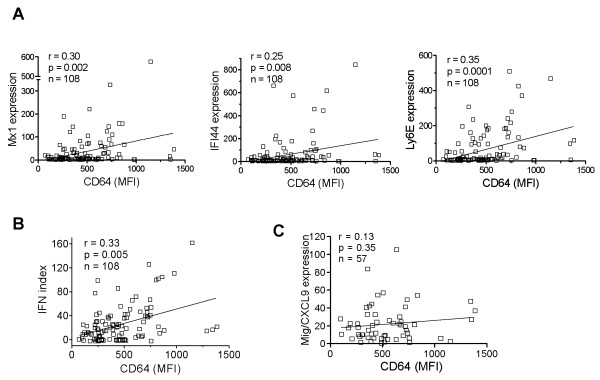
**CD64 expression correlates with the interferon signature in SLE**. Bivariate analysis of CD64 expression on monocytes (MFI, determined with flow cytometry; n = 108) and **(a) **the expression of the ISGs MX1, IFI44, and Ly6E (determined with real-time PCR), **(b) **the composite IFN score derived from the three ISGs, or **(c) **expression of the IFN-γ-inducible gene CXCL9. Spearman's correlation was used for all analyses.

Next we asked whether surface CD64 expression is related to IFN-I levels *in vivo*. Because ISG expression reflects serum IFN-I levels, we compared surface CD64 levels on monocytes with the transcript levels of three ISGs (MX1, IFI44, and Ly6E) in PBMCs from lupus patients (n = 108). The MFI of CD64 staining on monocytes correlated with the expression of each of these ISGs (Figure 3a; *P *< 0.01 for all comparisons, Spearman's rank correlation) as well as with the composite IFN index derived from the three ISGs (Figure 3b; *P *= 0.005).

IFN-γ is also a potent inducer of CD64 expression [24,25]. To address the potential involvement of IFN-γ, we compared CD64 expression with the transcript levels of CXCL9, a chemokine strongly induced by IFN-γ but only weakly by IFN-I [4]. No correlation was found between CD64 staining and CXCL9 expression (Figure 3c). Taken together, these data suggest that surface CD64 expression on monocytes from SLE patients reflects primarily IFN-I exposure.

### SLE serum induces surface expression of CD64 on monocytes

**Figure 4:**
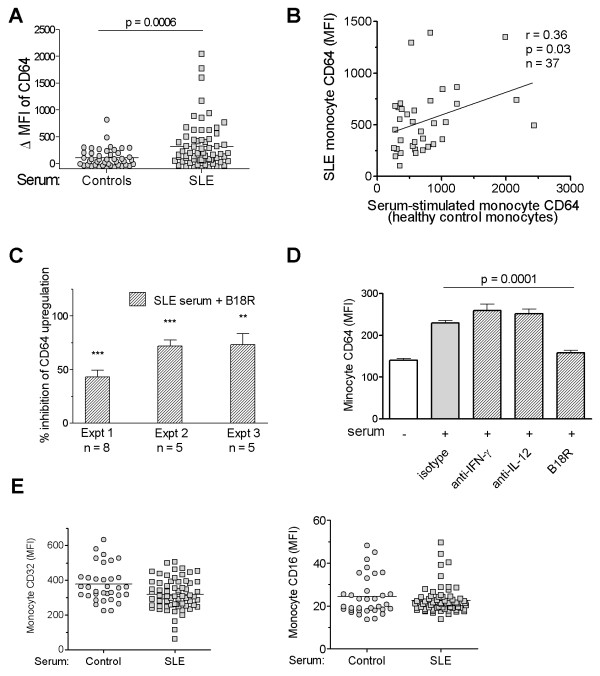
**SLE serum induces surface expression of CD64 on monocytes**. **(a) **Effect of sera from SLE patients (n = 65) and healthy controls (n = 44) on CD64 expression by healthy control monocytes. PBMCs from healthy donors were cultured in the presence of 25% serum for 19 hours before flow cytometry. ΔMFI was calculated by subtracting baseline CD64 MFI from donor monocytes cultured in autologous serum from the MFI of CD64 expression after incubation with serum from healthy controls or SLE patients. A positive ΔMFI indicates an upregulation of CD64 expression compared with the baseline levels. **(b) **Bivariate analysis of SLE serum-induced upregulation of CD64 on healthy control monocytes and CD64 expression on monocytes from the SLE serum donors (n = 37; *r *= 0.36; *P *< 0.05; Spearman's correlation). **(c) **Effect of B18R pretreatment on SLE serum-induced CD64 upregulation on healthy control monocytes. Three independent experiments, each using five or more serum samples from SLE patients are depicted. ** *P *< 0.01; *** *P *< 0.001 compared with the levels of CD64 induced by SLE-serum without B18R present (Student's *t *test). **(d) **Effect of anti-IFN-γ or anti-IL-12 neutralizing antibodies, or isotype control antibody (mouse IgG1) on SLE serum-induced upregulation of CD64 on healthy control monocytes. Bars represent the mean of four independent experiments. Difference in CD64 expression in the presence/absence of B18R was analyzed with Student's *t *test. **(e) **Flow-cytometry analysis of CD32 and CD16 expression on healthy control monocytes after incubation with sera from SLE patients or healthy controls, as described in (a).

Recently it was reported that IFN-I levels can be estimated by culturing an IFN-responsive cell line in the presence of SLE sera, by using the induction of ISG expression in the responder cells as a readout [26]. To examine whether IFN-I in SLE serum also induces monocyte CD64 expression, we cultured PBMCs from healthy donors overnight with serum samples from SLE patients (n = 65) or healthy controls (n = 44). As shown in Figure 4a, CD64 expression on monocytes increased significantly in the presence of SLE sera compared with sera from healthy controls (ΔMFI 319.7 ± 54.3 versus 104.3 ± 26.2; *P *< 0.001, Student's *t *test). It is noteworthy that sera from healthy controls also induced a mild upregulation of CD64 expression, although the difference was not statistically significant compared with effects of autologous sera from the monocyte donors (data not shown).

These data strongly suggest that one or more serum mediator(s) were responsible for the upregulation of CD64 expression on monocytes from SLE patients. Supporting this view, the ability of individual SLE sera to induce CD64 expression correlated with CD64 expression on monocytes from the serum donor (*r *= 0.36; *P *< 0.05, Spearman's rank correlation; Figure 4b). The effects of SLE sera were inhibited by the addition of B18R, but not neutralizing antibodies to IL-12 or IFN-γ, indicating that IFN-I was the major factor responsible for CD64 upregulation (Figure 4c and 4d). In contrast, the addition of SLE sera slightly decreased the expression of CD32 and had little effect on the expression of CD16 (Figure 4e). Taken together, these findings support the utility of CD64 as a biomarker of IFN-I dysregulation in SLE.

### Changes in CD64 expression after therapy

**Figure 5:**
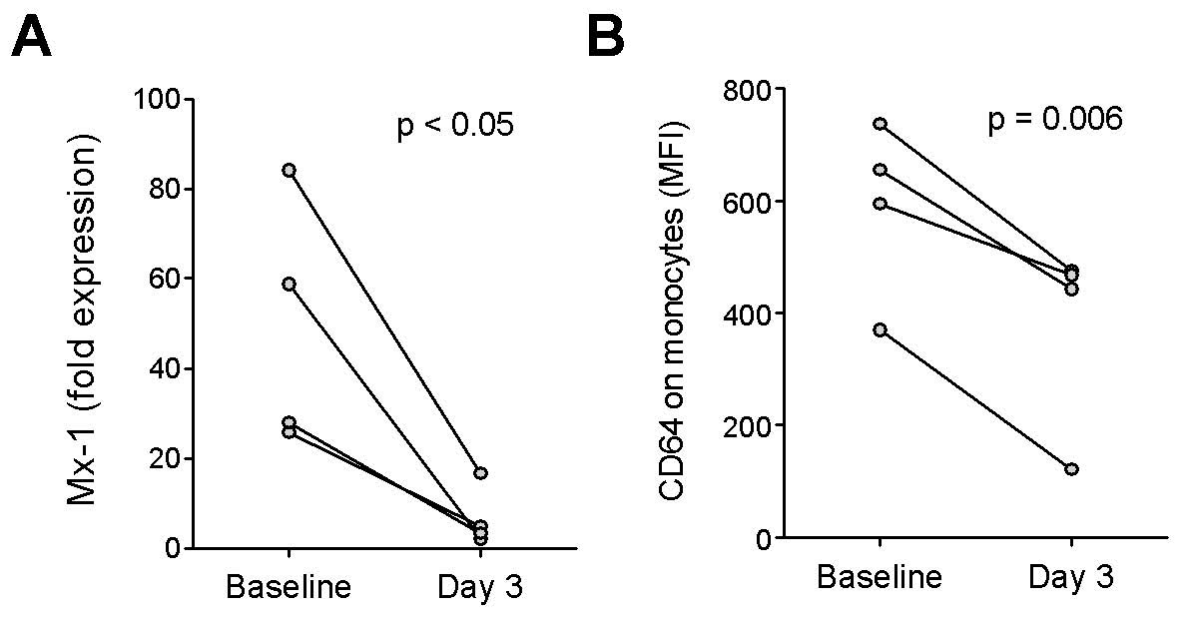
**Effect of therapy on CD64 fluorescence intensity**. **(a) **Expression of MX1 in PBMCs (measured with real-time PCR) and **(b) **surface expression of CD64 on monocytes (measured with flow cytometry) in four patients before (day 0) and after (day 3) therapy with high-dose methylprednisolone (1 g IV daily for 3 days). Differences were analyzed by using Student's paired *t *test.

Interferon dysregulation in SLE patients generally is unaffected by conventional medications, such as low-dose corticosteroids, antimalarials, and cytotoxic agents [5]. Only treatment with high-dose (pulse) corticosteroids seems to be effective in eliminating the interferon signature [1]. Consistent with this observation, we previously reported that CD64 expression on monocytes is largely unaltered by a daily corticosteroid dosage <40 mg [14]. We therefore examined whether CD64 expression can be used to monitor changes in IFN-I levels associated with high-dose corticosteroid therapy. In four SLE patients pulsed with high-dose methylprednisolone (1 g IV daily for 3 days), expression of the ISG MX1 was reduced significantly in PBMCs after treatment (*P *< 0.05; paired *t *test; Figure 5a). Supporting the utilization of CD64 as a biomarker of IFN-I levels, a concomitant reduction of CD64 expression on monocytes, but little effect on CD16 or CD32 expression was observed in all four patients (*P *< 0.006; paired *t *test; Figure 5b).

## Discussion

Elevated serum IFN-α was first noted in SLE patients about two decades ago [27]. More recently, high levels of ISG expression in lupus PBMCs have been widely reported [1-5]. This "interferon signature" can be identified by using microarrays or real-time PCR. These approaches have limitations, including the time and labor required to prepare and handle mRNA from clinical samples and their expense. Here, we evaluated the utility of CD64 as a marker for rapidly assessing IFN-I overproduction. Initial suggestions that CD64 is an ISG arose from microarray studies [2,16], although it remained unknown whether the increased gene expression was associated with changes at the protein level. Our data show for the first time that CD64 surface-staining intensity on monocytes correlates with ISG expression and disease manifestations in lupus patients and illustrate the potential utility of CD64 measurements for quantifying IFN-I levels in serum or other biologic fluids. We also show that CD64 may be used to monitor the effect of therapy on IFN-I levels.

Compared with real-time PCR and microarrays, CD64 expression on circulating monocytes provides a quick and relatively inexpensive means of assessing a patient's interferon status. One-step staining of whole-blood samples and analysis by using a standard four-color flow cytometer can be completed within 30 to 45 minutes, allowing results to be generated during a patient's clinic visit. Moreover, because MFI from flow cytometry staining is an absolute value, this assay avoids the need to normalize the data to actin or other housekeeping genes, as in real-time PCR assays.

The specificity of CD64 fluorescence intensity for IFN-I levels is suggested by several lines of evidence. CD64 expression was enhanced in a dose-dependent manner by IFN-α and was blocked by the viral IFN-I inhibitor B18R (Figure 2a). Similarly, TLR7 and TLR9 ligands enhanced monocyte surface CD64 expression in an IFN-I-dependent manner (Figure 2d). The ability of SLE sera to induce CD64 expression also depended on the presence of IFN-I. CD64 seems unique among the Fc receptors in its regulation by IFN-I. Although IFN-γ can also induce CD64 expression [25], its contribution to the interferon signature in SLE may be limited, as genes specifically induced by IFN-γ (that is, CXCL9) are not known to be upregulated in lupus patients [26]. In contrast to the lack of a correlation between CD64 fluorescence intensity and CXCL9 expression, surface CD64 expression correlated with the transcript levels of several ISGs (MX1, IFI44, and Ly6E) (Figure 3). In addition, upregulation of monocyte CD64 in the presence of lupus serum was inhibited by the poxviral B18R protein (Figure 4), strongly suggesting that IFN-I in SLE sera upregulates CD64 expression. This effect was not seen with blockade of IFN-γ or IL-12 with neutralizing antibodies, despite of the ability of these cytokines to induce CD64 expression [14].

Dysregulated CD64 expression may have functional consequences, as this activating FcγR plays important roles in phagocytosis, cytolysis, and induction of inflammatory cytokines [28]. The balance of activating (CD16, CD32a, CD64) and inhibitory (CD32b) FcγRs on antigen-presenting cells, such as monocytes, determines the response to immune complexes, which are produced abundantly in SLE. In addition, CD64 also is involved in the inflammatory response induced by C-reactive protein [29]. Elevated expression of CD64 in SLE, therefore, may fuel the chronic inflammation associated with the autoimmune disease. This view is supported by animal studies, as the deletion of Fc receptor γ-chain, a critical signaling component of the activating FcγRs, is sufficient to inhibit the development of glomerulonephritis lupus-prone mice [30]. The presence of Fc receptor γ-chain on monocytes/macrophages is required for this effect [31]. Besides monocytes, neutrophils, certain dendritic cell subsets (especially myeloid dendritic cells), and eosinophils also express surface CD64 [32]. In particular, the fluorescence intensity of CD64 on myeloid dendritic cells from lupus patients was increased and correlated highly with that on monocytes (data not shown). However, because of the paucity of circulating DCs in most SLE patients [5], they are technically difficult to analyze.

Some limitations to the clinical application of CD64 expression merit consideration, notably in patients with infections. Neutrophil CD64 has been used as a marker for sepsis [32-35] and to distinguish between infections with dsDNA and ssRNA viruses [35,36]. It has been suggested that neutrophil CD64 expression can be used to aid in the diagnosis of infections in patients with rheumatoid arthritis [37]. Conversely, in the setting of sepsis, the existence of preexisting autoimmune disease, especially lupus, is a potential confounder. In addition, although CD64 appears to be a surrogate marker of IFN-I activity based on our cross-sectional analysis, longitudinal studies are needed to assess the utility of this marker in monitoring changes in serum IFN-I levels over time.

Recently it was reported that Siglec-1 (CD169) expression on monocytes can be used as a biomarker for IFN-I responses in systemic sclerosis and SLE [16,38,39]. Compared with the two-step flow-cytometry assay for CD169, measuring CD64 on circulating monocytes is simpler, requiring only a single step. Whether CD169 is suitable for bioassays using serum samples has also not been tested. However, similar to the desirability of measuring the expression of more than one ISG with real-time PCR, the use of both CD64 and CD169 staining may be warranted to optimize the reliability of the assay.

## Conclusions

Our results indicate that the fluorescence intensity of CD64 on circulating monocytes can be used to evaluate IFN-I levels. This flow-cytometry assay is faster and less labor intensive than the measurement of ISG gene expression. As novel agents targeting IFN-I are already in clinical trials [40], flow-cytometry analysis of CD64 expression may be a convenient and rapid approach for estimating IFN-I levels in SLE patients.

## Abbreviations

ACR: American College of Rheumatology; C3, C4: complement component 3 and complement component 4; FcγR: Fc gamma receptor; hs-CRP: high-sensitivity C-reactive protein; IC: immune complex; IFN-I: type I interferon; ISG: interferon-stimulated gene; LN: lupus nephritis; LPS: lipopolysaccharide; MDCs: myeloid dendritic cells; MFI: mean fluorescence intensity; PBMCs: peripheral blood mononuclear cells; SLE: systemic lupus erythematosus; SLEDAI: SLE disease activity index; TLR: Toll-like receptor.

## Competing interests

The authors declare that they have no competing interests.

## Authors' contributions

YL and WHR contributed to the study design. YL, EK, MP, YX, JS, HZ, ESB, MSS, SM, and WHR contributed to the acquisition of data. YL, PYL, and WHR contributed to the analysis and interpretation of data. YL, PYL, and WHR contributed to manuscript preparation. YL contributed to statistical analysis.
